# Molecular Detection of *Toxoplasma gondii*, *Neospora caninum* and *Encephalitozoon* spp. in Vespertilionid Bats from Central Europe

**DOI:** 10.3390/ijms24129887

**Published:** 2023-06-08

**Authors:** Eva Bártová, Jiřina Marková, Jana Sedláčková, Hana Banďouchová, Karol Račka

**Affiliations:** 1 Department of Biology and Wildlife Diseases, Faculty of Veterinary Hygiene and Ecology, University of Veterinary Sciences Brno, Palackého tř. 1946/1, 61242 Brno, Czech Republic; bartovae@vfu.cz (E.B.); jirina.markova@gmail.com (J.M.); 2 Department of Ecology and Diseases of Game, Fish and Bees, Faculty of Veterinary Hygiene and Ecology, University of Veterinary Sciences Brno, Palackého tř. 1946/1, 61242 Brno, Czech Republic; jsedlackova@vfu.cz (J.S.); bandouchovah@vfu.cz (H.B.); 3 Department of Epizootology, Parasitology and Protection of One Health, University of Veterinary Medicine and Pharmacy in Košice, Komenského 73, 04181 Košice, Slovakia

**Keywords:** insectivore, microsporidiosis, molecular methods, neosporosis, toxoplasmosis

## Abstract

Bats may carry various viruses and bacteria which can be harmful to humans, but little is known about their role as a parasitic source with zoonotic potential. The aim of this study was to test wild bats for the presence of selected parasites: *Toxoplasma gondii*, *Neospora caninum* and microsporidia *Encephalitozoon* spp. In total, brain and small intestine tissues of 100 bats (52 *Myotis myotis*, 43 *Nyctalus noctula* and 5 *Vespertilio murinus*) were used for the DNA isolation and PCR detection of the abovementioned agents. *Toxoplasma gondii* DNA was detected by real-time PCR in 1% of bats (in one male of *M. myotis*), while all bats were negative for *N. caninum* DNA. *Encephalitozoon* spp. DNA was detected by nested PCR in 25% of bats, including three species (twenty-two *M. myotis*, two *N. noctula* and one *V. murinus*). Positive samples were sequenced and showed homology with the genotypes *Encephalitozoon cuniculi* II and *Encephalitozoon hellem* 2C. This is the first study on wild vespertilionid bats from Central Europe and worldwide, with a relatively high positivity of *Encephalitozoon* spp. detected in bats.

## 1. Introduction

The Vespertilionidae family (vespertilionid bats) belongs to the Chiroptera order, which is the second largest mammalian order with an almost worldwide distribution. Microchiroptera, its suborder, comprises approximately one-third of European mammalian species [1]. Vespertilionid bats are very diverse in size, morphology, food and composition. Besides their natural habitats, they may live and roost within urban areas [2]. Since they can come into contact with humans, there is a question of the occurrence of pathogens and their transmission to humans and within the bat species [3,4].

Bats are well known to have a special relationship with viruses [5]. In databases of bat viruses, there are now about 1353 viruses described in bats in Europe [6]. After several outbreaks of infections with SARS-coronavirus, Ebola virus, Nipah virus and Hendra virus, bats gained increased attention as a potential reservoir host [6]. Bacteria (such as *Bartonella* spp. and *Borrelia* spp.) have also been detected in bats. In recent years, bat-associated *Bartonella* genotypes have been found in humans, indicating the public health importance of this bacteria in bats [7]. Recently, little is known about the occurrence of protozoan parasites in European bats and that is why the aim of our study was to detect the DNA of *Toxoplasma gondii* (Nicolle and Manceaux, 1908), *Neospora caninum* (Dubey, Carpenter, Speer, Topper and Uggla, 1988) and *Encephalitozoon* spp. in vespertilionid bats coming from Central Europe. We expect that due to the wide diversity and different living conditions of Chiroptera, the spectrum of ecto- and endoparasites may vary between individual bat species [1,8,9,10].

## 2. Results

*Toxoplasma gondii* DNA was detected in 1% of bats, in the brain of one male greater mouse-eared bat *Myotis myotis* (Borkhausen, 1797) found dead in the South Moravian region of the Czech Republic. Genotyping of *T. gondii* positive sample was not successful. All bats were negative for *N. caninum.* Microsporidian DNA was detected in 25% of bats, including twenty-two *M. myotis*, two common noctule *Nyctalus noctula* (Schreber, 1774) and one parti-colored bat *Vespertilio murinus* (Linnaeus, 1758), all from the Czech Republic (Supplemental Figure S1). There was no statistical difference (*p* = 0.8174) of prevalence between genders of bats (26% in males and 24% in females), but there was a statistical difference in positivity between localities (*p* = 0.002). Scheffé’s method showed a difference between Bohemia and Moravia (*p* = 0.0134) and between Moravia and the Slovak Republic (*p* = 0.0181). Six positive samples were successfully sequenced, showing homology with *Encephalitozoon cuniculi* (Levaditi, Nicolau & and Schoen, 1923) genotype II in two *N. noctula* from the Czech Republic (Prague and Central Bohemian region) and in one *V. murinus* from the Czech Republic, and *Encephalitozoon hellem* (Didier, Didier, Friedberg, Stenson, Orenstein, Yee, Tio, Davis, Vossbrinck, Millichamp and Shadduck, 1991) genotype 2C in three *M. myotis* (two from the South Moravian and Central Bohemian regions and one from the Moravian-Silesian region) (Table 1).

## 3. Discussion

The first molecular detection of *T. gondii* by nested PCR was described in the synanthropic bat species Pallas’s free-tailed bat *Molossus molossus* (Pallas, 1766), common vampire bat *Desmodus rotundus* (Geoffroy, 1810) and Pallas’s long-tongued bat *Glossophaga soricina* (Pallas, 1766) from Brazil [11]. Sun et al. [12] detected *T. gondii* by a nested PCR in 29% of 550 insectivorous species of bats found in Myanmar, Asia. Other similar studies on different bat species showed a relatively low *T. gondii* positivity. Dodd et al. [13] described a 10% positivity in the insectivorous species of common pipistrelle *Pipistrellus pipistrellus* (Schreber, 1774) and soprano pipistrelle *Pipistrellus pygmeus* (Leach, 1825) from Western Europe, tested by nested PCR. De Jesus et al. [14] detected *T. gondii* by conventional PCR in 2% of Seba’s short-tailed bat *Carollia perspicillata* (Linnaeus, 1758) from Brazil, while all tested bats were negative for *N. caninum*. To date, the parasite *N. caninum* was only detected in the brain of one fruit and three insectivorous bats from China by nested PCR [15]. In our study, we proved the presence of *T. gondii* in only one male *M. myotis*, found close to the human residences in the Czech Republic. *N. caninum* was not detected in any of the examined bats. According to our findings, vespertilionid bats can probably serve as an intermediate host for *T. gondii,* while the presence of *N. caninum* in the European bat species is still questionable. Bats from the Vespertilionidae family are insectivorous animals, which raises the question of acquiring the *T. gondii* infection. The only positive individual was the *M. myotis*, whose diet consists mainly of ground beetles of *Carabus* and *Pterostichus* [16]. That is why we hypothesize that the *T. gondii* infection was presumably acquired via accidental ingestion of an oocyst from the soil, water drops or insect prey as a mechanical carrier. Studies from China and Brazil confirmed the presence of *T. gondii* in various tissues, such as the brain, heart and striated muscles, by nested PCR [17,18]. In our study, *T. gondii* DNA was isolated from the brain tissue of one *M. myotis* individual, whereas other tissues were not tested. According to the results from previous studies, we can also assume the occurrence of *T. gondii* tissue cysts in the heart and muscles of the *M. myotis* from our study. However, nested PCR was used in most cited studies for the detection of both *T. gondii* and *N. caninum* DNA. In our study, we used qPCR, targeting the 529 bp repeat element of *T. gondii* DNA, which is considered as a highly sensitive method that is able to detect one *T. gondii* genome equivalent (80 fg DNA) per sample [19]. We recommend using the qPCR targeting appropriate genes of *N. caninum* in further studies that might show a higher positivity in bats.

Microsporidia *Encephalitozoon* spp. has a zoonotic potential and, apart from a wide spectrum of mammalian and avian species, can also infect humans. There are many studies concerning *Encephalitozoon* spp. in wildlife, but the evidence of this infection in bats is still missing. Lallo et al. [20] detected microsporidia spores in the feces of wild hematophagous hairy-legged vampire bat *Diphylla ecaudata* (Spix, 1823) from Brazil. Another similar microsporidia *Enterocytozoon bieneusi* (F. Desp., Le Charpentier, Galian, Bernard, Cochand-Priollet, Lavergne, Ravisse and Modigliani, 1985) of six different genotypes were proven in the intestines of 5.2% of bats and in the feces of 1.9% of bats from South Korea [21]. The spores secreted into the environment could be the source of infection in other mammals and birds. In our study, we detected *E. cuniculi* genotype II in two *N. noctula* and *E. hellem* genotype 2C in three *M. myotis.* These genotypes were described in many wild and domestic animals, with *E. hellem* having been proven especially in birds from Europe and other countries worldwide [22]. Childs-Sanford et al. [23] described one case of fatal microsporidiosis with renal and liver alterations caused by *E. hellem* in a captive female of the Egyptian fruit bat *Rousettus egyptiacus* (E. Geoffroy, 1810) from the New York Zoo. *E. cuniculi* and *E. hellem* can also rarely cause human infection especially in immunocompromised patients where disseminated ocular, respiratory and urogenital infections have been described [24,25]. The bats positive for *Encephalitozoon* spp. in our study were without any visible lesions in the dissected tissues.

Bats were identified as natural reservoir hosts for several emerging viruses that can induce severe disease in humans, including RNA viruses such as Marburg virus, Hendra virus, Sosuga virus and Nipah virus [26]. Since the COVID-19 crisis, calls for an integrated One Health surveillance as a key to pandemic prevention and preparedness was highlighted and reemphasized by many scientists and experts. A typical example of One Health approach was implemented in Vietnam, illustrating the high risk for CoV spillover from bats to pigs [27]. Regarding fungal pathogens, a One Health approach pilot study was carried out in Mpumalanga Province, the Republic of South Africa in 2018. The analysis of fecal samples revealed that bats can harbor pathogenic fungi such as *Aspergillus fumigatus* (Fresenius 1863) and *Aspergillus flavus* (Link 1809) that cause human diseases [28]. Very few studies focused on the parasitic pathogens of bats. High diversity of *Trypanosoma* species were revealed in bats, including species with medical and veterinary importance such as *Trypanosoma cruzi* (Chagas, 1909) and *Trypanosoma evansi* (Steel Chauvrat, 1896). One Health approach was used to discuss the significance of bats as reservoirs of *T. cruzi* [29]. The importance of parasites associated with bats investigated in our study in One Health approach is still unknown. However, bats are consumed by humans in several regions of Africa, Asia and Oceania [30,31,32]. Consumption of raw or undercooked bat meat may present a certain risk of *T. gondii* in humans. Bats are also considered as important reservoirs for various emerging viruses, presumably also involving the SARS-CoV-2 virus [33,34,35]. There are also several studies presuming that the coinfection of *T. gondii* and SARS-CoV-2 virus may deteriorate the clinical outcome in patients with COVID-19 infection [36,37]. As a result, bats may present a doubled risk in countries using these animals as part of their cuisine.

To our knowledge, this is the first study on wild vespertilionid bats from Central Europe and worldwide, with a relatively high positivity of *Encephalitozoon* spp. in bats. The source of protozoal oocyst and microsporidian spores for wild bats is unknown. The bats can most likely come into contact with parasites during roosting, feeding and also by drinking contaminated water. Because of their good adaptation to urban places and living in old houses and churches, the risk of direct contact with definitive hosts or vectors (e.g., cats, dogs, rodents, birds) of the infection is higher, compared to the other groups of bats. Our results show that members of the Vespertilionidae family can serve as intermediate hosts of protozoan parasites and come into contact with the spores of microsporidia. Thus, infected bats are the potential source of infection for their colony, predators and for humans. Except for a large number of viruses with zoonotic potential, bats may also harbor different protozoan species that may present a certain risk for animals and humans. Further investigations focusing on larger group of parasites and other zoonotic pathogens and more sensitive diagnostic tools such as NGS-based analysis should be addressed. In terms of One Health cross-sectoral surveillance approach, various biological samples from bat–human contact sites should be targeted.

## 4. Material and Methods

### 4.1. Tested Animals

In total, 100 bats from the Vespertilionidae family (52 *M. myotis*, 43 *N. noctula* and 5 *V. murinus*) were used in our study. The animals were found dead, close to the human residences or in the natural surroundings in the Czech Republic and the Slovak Republic between the years 2010 and 2017 (Supplementary Figure S2, Table 2). Bat carcasses from the Czech Republic (n = 90) were provided by bat researchers and wildlife rehabilitation centers. Bat carcasses from the Slovak Republic (n = 10) were provided by bat rehabilitation centers, but unfortunately without any information on their localities. The animals were sorted according to species and gender (50 males and 50 females) and dissected. Pieces of the brain and small intestine tissues were collected and frozen at −20 °C until assayed.

### 4.2. Methods of Molecular Biology

The tissues of bats (brain and small intestine) were homogenized in tubes with ceramic marbles, using the MagNA Lyser Instrument tissue homogenizer (Roche, Basel, Switzerland) at a rotation speed of 6500 rpm/2× 45 s. Thereafter, the DNA was isolated, using the DNeasy^®^ Blood and Tissue Kit (Qiagen, Hilden, Germany) according to the manufacturer’s instructions.

The PCR reaction was performed in a 25 µL volume, containing the isolated DNA, a commercial premix—PPP Master Mix (Top-Bio s.r.o., Prague, Czech Republic), PCR-grade water and specific primers. Negative and positive controls were included in each reaction.

Brain samples were tested for *T. gondii* DNA by real-time PCR, targeting a 529 bp repeated fragment of *T. gondii* DNA, as previously described [38], using primers TOX4 and TOX5 (Table 2). Positive samples were proceeded to genotyping, using 15 microsatellite markers in multiplex PCR (Table 3) and automatic sequencer electrophoresis (ABI PRISM 3130x1; Applied Biosystems) [39]. The results were analyzed with GeneMapper software (version 4.0; Applied Biosystems) to estimate the size of alleles (base pairs).

Brain tissues were also used for the detection of *N. caninum* DNA using a single PCR with primers Np6+ /Np21+ annealing to the Nc-5 region [40], respectively (Table 2). The PCR protocol for the detection of *N. caninum* consisted of 94 °C for 5 min, 35 cycles of amplification (94 °C for 1 min, 63 °C for 30 s and 72 °C for 1 min) and 72 °C for 10 min.

Small intestine tissues were used for the detection of microsporidia *Encephalitozoon* spp. DNA using a nested PCR with two different MSP primer pairs (MSP1, MSP2A and MSP3, MSP4A) targeting the ITS region [41] (Table 2). Both nested PCR reactions consisted of 92 °C for 2 min, followed by 35 cycles (92 °C for 1 min, 59 °C for 1 min and 72 °C for 1.5 min) and 72 °C for 5 min. The PCR products were analyzed under a UV light, after electrophoresis on 1.5 % agarose gel stained with Midori green. The molecular weight of PCR products was 317 bp for *N. caninum* and 300 bp for *Encephalitozoon* spp. The positive microsporidian PCR products were purified by a Gel/PCR Fragments Extraction Kit (Geneaid, New Taipei City, Taiwan) and sent for sequencing in both directions (Macrogen company, Amsterdam, the Netherlands). Final sequences were aligned, edited by Staden Package Programs Pregap4 and Gap4 and compared with the GenBank database via BLAST (basic local alignment search tool) search (https://blast.ncbi.nlm.nih.gov/Blast/, accessed on 20 September 2022).

### 4.3. Statistical Analysis

The results were statistically analyzed with Pearson’s chi-square test or Fisher exact test for independence using STATISTICA Cz 12 [42] and RStudio Version 1.2.5033. We tested the null hypothesis that the presence of selected parasites *T. gondii*, *N. caninum* and microsporidia *Encephalitozoon* spp. in tissues of vespertilionid bats does not differ in localities and gender. The differences were considered statistically significant if the *p*-value was <0.05. In the case of a statistically significant difference of localities, the Scheffé multiple comparison method [42] was subsequently applied. A locality for which no information was known was excluded from the analysis.

## Figures and Tables

**Table 1:** Vespertilionid bats species examined for *Encephalitozoon* spp., sorted according to localities and gender.

Characteristic	Animal Species			Total
	*Myotis myotis*	*Nyctalus noctula*	*Vespertilio murinus*	
Localities	*p* = 0.0409	*p* = 0.2258	*p* > 0.9999	
Czech Republic	22/50 (44%)	2/35 (6%)	1/5 (20%)	25/90 (28%)
Central Bohemian	1/5 (20%)	0/10 (0%)	1/2	2/17 (12%)
Moravian-Silesian	1/7 (14%)	–	–	1/7 (14%)
Plzeň	0/1	0/2	–	0/3
Prague	–	1/5 (20%)	0/1	1/6 (17%)
South Moravia	19/32 (59%)	0/6 (0%)	0/2	19/40 (48%)
No information	1/5 (20)	1/12 (8%)	0	2/17 (12%)
Slovak Republic	0/2	0/8 (0%)	0/0	0/10 (0%)
Gender	*p* > 0.5234	*p* > 0.9999	*p* = 0.4000	
Female	10/21 (48%)	1/27 (4%)	1/2	12/50 (24%)
Male	12/31 (39%)	1/16 (6%)	0/3	13/50 (26%)
Total	22/52 (42%)	2/43 (5%)	1/5 (20%)	25/100 (25%)

In total, two genotypes were characterized in bats from the Czech Republic: genotype *E. cuniculi* II was detected in two *N. noctula* and in one *V. murinus* and genotype *E. hellem* 2C was detected in three *M. myotis.*

**Table 2:** Primers used in PCR to detect *Encephalitozoon* spp., *Neospora caninum* and *Toxoplasma gondii* in bats collected in the Czech Republic and the Slovak Republic.

Primer Name	Oligonucleotide Sequences
*Encephalitozoon cuniculi* (ITS region)	
MSP 1	5′ TGA ATG (G,T)GT CCC TGT 3′
MSP 2A	5′ TCA CTC GCC GCT ACT 3′
MSP 3	5′ GGA ATT CAC ACC GCC CGT C(A,G)(C,T) TAT 3′
MSP 4A	5′ CCA AGC TTA TGC TTA AGT (C,T)(A,C)A A(A,G)G GG 3′
*Neospora caninum* (Nc-5 region)	
Np21+	5′ CCCAGTGCGTCCAATCCTGTAAC 3′
Np6+	5′ CTCGCCAGTCAACCTACGTCTTCT 3′
*Toxoplasma gondii* (529-bp repeated element)	
TOX4	5′ CTGCAGGGAG GAAGACGAAA GTTG 3′
TOX5	5′ CGCTGCAGACACAGTGCATCTGGATT 3′

**Table 3:** Microsatellite markers and primers used for *Toxoplasma gondii* genotyping by multiplex PCR assay.

Marker	Primer Sequence
TUB2	5′ 6-FAM-GTCCGGGTGTTCCTACAAAA 3′
	5′ TTGGCCAAAGACGAAGTTGT 3′
W35	5′ HEX-GGTTCACTGGATCTTCTCCAA 3′
	5′ AATGAACGTCGCTTGTTTCC 3′
TgM-A	5′ HEX-GGCGTCGACATGAGTTTCTC 3′
	5′ TGGGCATGTAAATGTAGAGATG 3′
B18	5′ 6-FAM-TGGTCTTCACCCTTTCATCC 3′
	5′ AGGGATAAGTTTCTTCACAACGA 3′
B17	5′ HEX-AACAGACACCCGATGCCTAC 3′
	5′ GGCAACAGGAGGTAGAGGAG 3′
M33	5′ 6-FAM-TACGCTTCGCATTGTACCAG 3′
	5′ TCTTTTCTCCCCTTCGCTCT 3′
IV.1	5′ HEX-GAAGTTCGGCCTGTTCCTC 3′
	5′ TCTGCCTGGAAAAGGAAAGA 3′
XI.1	5′ 6-FAM-GCGTGTGACGAGTTCTGAAA 3′
	5′ AAGTCCCCTGAAAAGCCAAT 3′
M48	5′ 6-FAM-AACATGTCGCGTAAGATTCG 3′
	5′ CTCTTCACTGAGCGCCTTTC 3′
M102	5′ NED-CAGTCCAGGCATACCTCACC 3′
	5′ CAATCCCAAAATCCCAAACC 3′
N60	5′ NED-GAATCGTCGAGGTGCTATCC 3′
	5′ AACGGTTGACCTGTGGCGAGT 3′
N82	5′ HEX-TGCGTGCTTGTCAGAGTTC 3′
	5′ GCGTCCTTGACATGCACAT 3′
AA	5′ NED-GATGTCCGGTCAATTTTGCT 3′
	5′ GACGGGAAGGACAGAAACAC 3′
N61	5′ 6-FAM-ATCGGCGGTGGTTGTAGAT 3′
	5′ CCTGATGTTGATGTAAGGATGC 3′
N83	5′ 6-FAM-ATGGGTGAACAGCGTAGACA 3′
	5′ GCAGGACGAAGAGGATGAGA 3′

## Data Availability

Data are available upon request from the authors.

## Supplementary Materials

The supporting information can be downloaded at: https://www.mdpi.com/article/10.3390/ijms24129887/s1.
