# Supplementary material for: Molecular Detection of Toxoplasma gondii, Neospora caninum and Encephalitozoon spp. in Vespertilionid Bats from Central Europe
Source: Int J Mol Sci. 2023 Jun 8;24(12):9887. doi: 10.3390/ijms24129887 (PMC10298469; doi:10.3390/ijms24129887)
Supplement: Supplementary file 1 [file ijms-24-09887-s001.zip › ijms-2396779-supplementary.pdf]

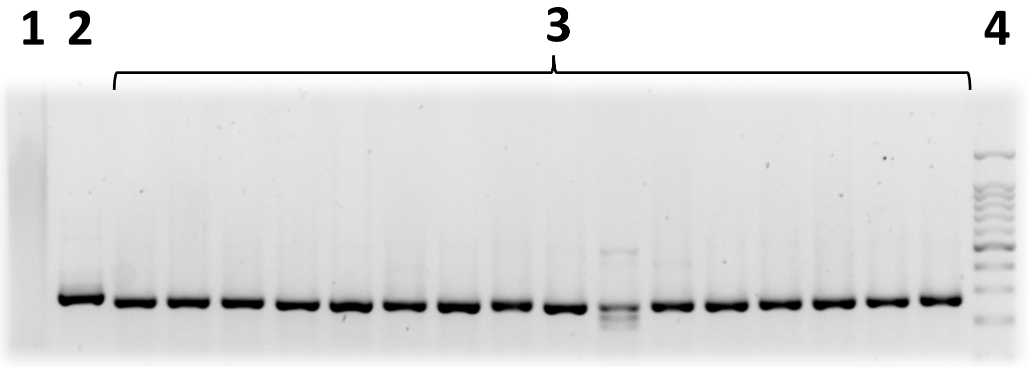

Supplemental Figure S1. Example of agarose gel with products of the second reaction from nested PCR to detect *Encephalitozoon cuniculi* in tissue of bats. (1) negative control (PCR grade water), (2) positive control 300 bp (DNA from spores of *E. cuniculi*) (3) *M. myotis* samples positive to *E. cuniculi,* (4) ladder (100 bp)

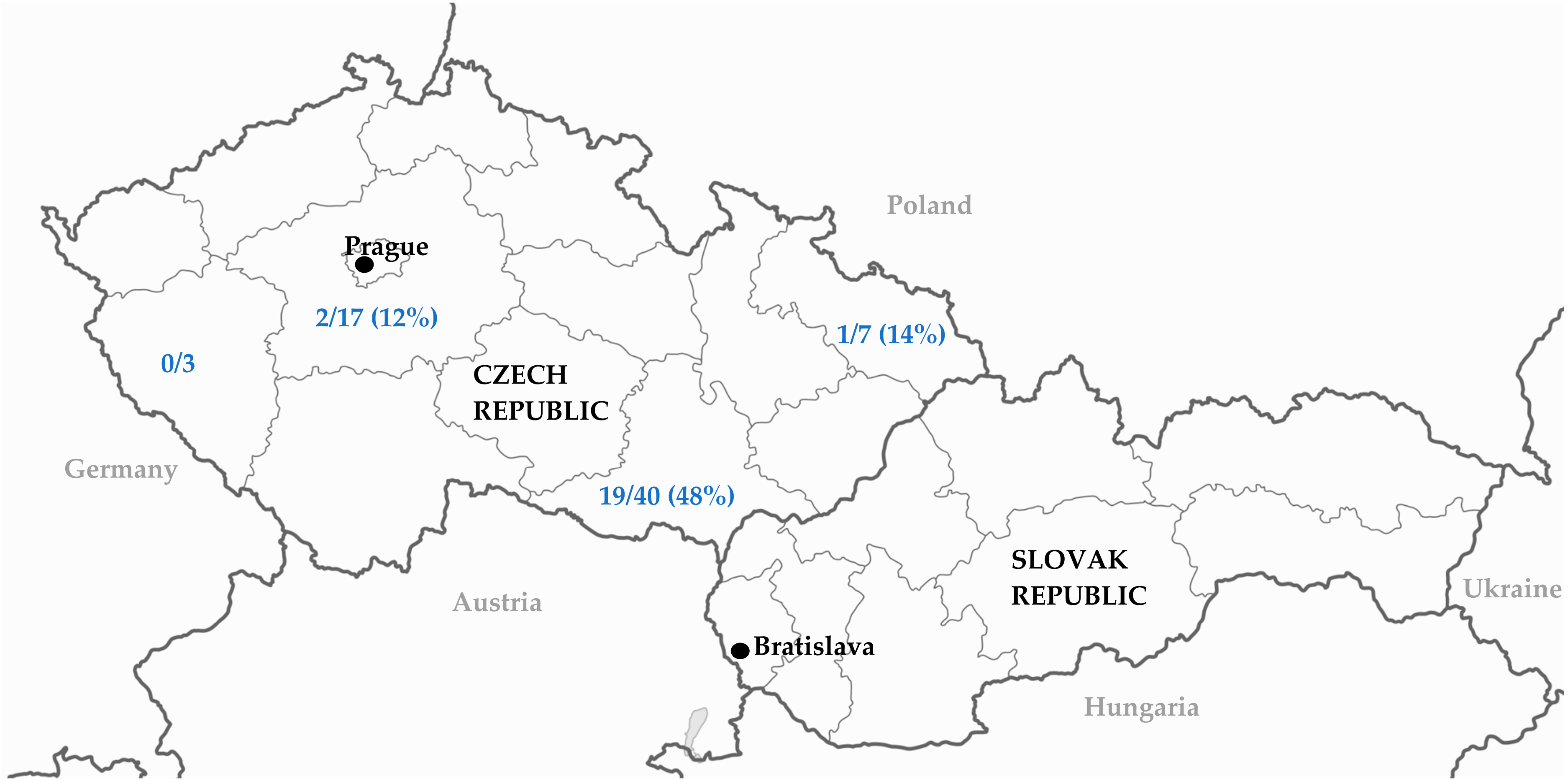

Supplemental Figure S2. The map of the Czech Republic and the Slovak Republic showing localities where bats (52 *Myotis myotis*, 43 *Nyctalus noctula* and 5 *Vespertilio murinus*) were collected with the prevalence of *Encephalitozoon cuniculi*: Central Bohemian 12% (2/17), Moravian-Silesian 14% (1/7), Plzeň (0/3), South Moravia 48% (19/40), the Czech Republic with no information about locality 12% (2/17), the Slovak Republic with no information about locality (0/10).
